# 1′,3′,3′-Trimethyl-2,3-diphenyl-2,3-di­hydro­isoxazole-5(4*H*)-spiro-2′-indoline

**DOI:** 10.1107/S1600536809002062

**Published:** 2009-01-23

**Authors:** Naoual Laghrib, Jean-Claude Daran, Rachid Fihi, Lhou Majidi, Mohamed Azrour

**Affiliations:** a Laboratoire des Substances Naturelles & Synthèse et Dynamique Moléculaire, Faculté des Sciences et Techniques, BP 509, Errachidia, Morocco; b Laboratoire de Chimie de Coordination, UPR–CNRS 8241, 205 route de Narbonne, 31077 Toulouse Cedex, France; c Laboratoire de Physico-Chimie des Matèriaux, Faculté des Sciences et Techniques, BP 509, Errachidia, Morocco

## Abstract

Two diastereoisomers of the title compound, C_25_H_26_N_2_O, have been prepared by cyclo­addition between 1,3,3-trimethyl-2-methyl­eneindoline and *C*-phenyl-*N*-phenyl­nitrone. The stereochemistry of the major diastereoisomer, *viz. S,R*/*R,S*, is confirmed by the X-ray analysis. The oxazole and the pyrole rings have envelope conformations. The packing is stabilized by weak C—H⋯π inter­actions involving the phenyl ring attached to the N atom of the oxazole and the phenyl ring of the indole fragment.

## Related literature

For general background, see: Alonso-Perarnau *et al.* (1997 ▶); Cacciarini *et al.* (2000 ▶); Pariera *et al.* (1993 ▶). For related studies, see: Daran *et al.* (2006 ▶); Fihi *et al.* (1995 ▶, 2004 ▶); Roussel *et al.* (2000 ▶, 2003 ▶). For the synthetic procedure, see: Brüning *et al.* (1973 ▶). For puckering parameters, see: Cremer & Pople (1975 ▶). 
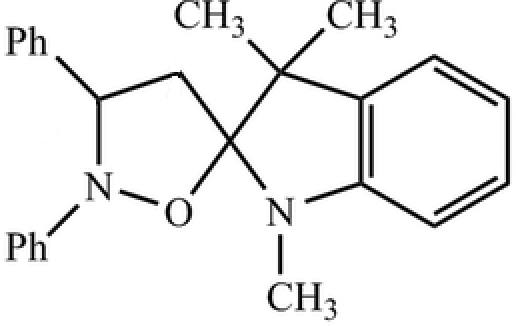


## Experimental

### 

#### Crystal data

C_25_H_26_N_2_O
*M* *_r_* = 370.48
Orthorhombic, 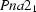

*a* = 18.0393 (18) Å
*b* = 8.9854 (7) Å
*c* = 12.3947 (9) Å
*V* = 2009.1 (3) Å^3^
*Z* = 4
Mo *K*α radiation
μ = 0.08 mm^−1^
*T* = 180 (2) K
0.48 × 0.36 × 0.28 mm

#### Data collection

Stoe IPDS diffractometer
Absorption correction: none
19030 measured reflections
2021 independent reflections
1581 reflections with *I* > 2σ(*I*)
*R*_int_ = 0.059

#### Refinement

*R*[*F* ^2^ > 2σ(*F* ^2^)] = 0.042
*wR*(*F* ^2^) = 0.105
*S* = 1.15
2021 reflections
256 parameters
1 restraint
H-atom parameters constrained
Δρ_max_ = 0.26 e Å^−3^
Δρ_min_ = −0.15 e Å^−3^

### 

Data collection: *IPDS* (Stoe & Cie, 2000 ▶); cell refinement: *IPDS*; data reduction: *X-RED* (Stoe & Cie, 1996 ▶); program(s) used to solve structure: *SIR97* (Altomare *et al.*, 1999 ▶); program(s) used to refine structure: *SHELXL97* (Sheldrick, 2008 ▶); molecular graphics: *ORTEPIII* (Burnett & Johnson, 1996 ▶) and *ORTEP-3 for Windows* (Farrugia, 1997 ▶); software used to prepare material for publication: *SHELXL97*.

## Supplementary Material

Crystal structure: contains datablocks I, global. DOI: 10.1107/S1600536809002062/fl2228sup1.cif

Structure factors: contains datablocks I. DOI: 10.1107/S1600536809002062/fl2228Isup2.hkl

Additional supplementary materials:  crystallographic information; 3D view; checkCIF report

**Fig. 1.**
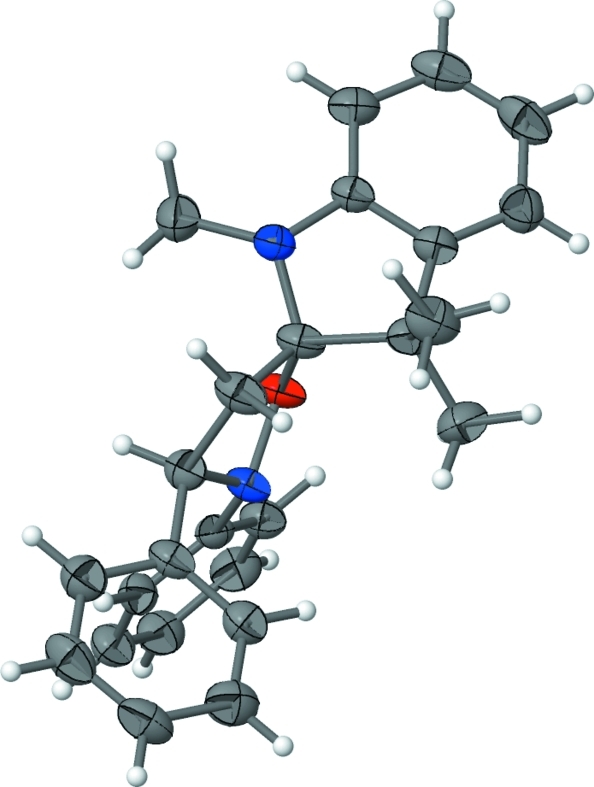
Molecular view of (I). Displacement ellipsoids are drawn at the 30% probability level and H atoms are represented as small spheres of arbitrary radii.

Enhanced figure: interactive version of Fig. 1

## Figures and Tables

**Table 1:** Hydrogen-bond geometry (Å, °) *Cg*1is the centroid of the C21–C26 ring and *Cg*2 is the centroid of the C3–C8 ring.

*D*—H⋯*A*	*D*—H	H⋯*A*	*D*⋯*A*	*D*—H⋯*A*
C7—H7⋯*Cg*1^i^	0.95	2.89	3.735 (3)	149
C23—H23⋯*Cg*2^ii^	0.95	2.95	3.803 (4)	150

Symmetry codes: (i) 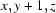; (ii) 
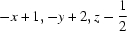
.

**Fig. 2.**
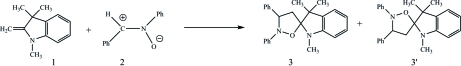
The formation of the title compound.

## supplementary crystallographic information

### Comment

Heterocyclic spirocompounds are of interest in synthetic organic chemistry (Pariera *et al.*, 1993; Alonso-Perarnau *et al.*, 1997; Cacciarini *et al.*, 2000). The cycloaddition between dipolarophiles bearing an exocyclic carbon-carbon double bond and appropriate1,3-dipoles is one of the best methods for the synthesis of bicyclic spirocompounds.

As part of our research on bicyclic spirocompounds (Fihi *et al.*, 1995; Roussel *et al.*, 2000, 2003; Daran *et al.*, 2006), we reported that methylene lactones react with 1,3-dipoles with high selectivity. In a previous article (Fihi *et al.*, 2004) we reported that 1,3-dipolar cycloaddition of arylnitriloxydes and *N*-Phenylarylnitrilimines to 5-chloro-2-methylene-1,3,3-trimethylindoline is regiospecific. Arylnitriloxydes reactions lead to spiroheterocyclic compounds, whereas *N*-phenylarylnitrilimines reactions afforded evolutives products.

We report here, the cycloaddition of *C*-phenyl-*N*-phenylnitrone (2) to 2-methylene-1,3,3-trimethylindoline (1). The reaction produced a mixture of diastereoisomers (Fig. 2). The ratio (77 / 23%) of which was evaluated by ^1^HNMR (performed on the crude reaction mixture). To confirm unambiguously the structure assignment of (3) and (3'), and to establish the stereochemistry of each spiroheterocycle, an X-ray structural analyses was carried out on the major spirocompound, because the ^1^H and ^13^CNMR studies did not provide unambiguous information.

The stereochemistry of the major diastereoisomer, *S,R*/*R,S*, is confirmed by the X-ray analyses (Fig. 1). The oxazole and the pyrole rings have an envelope conformation with puckering parameters Q(2)= 0.399 (3)°, φ(2)= 218.9 (5)° and Q(2)= 0.274 (3)°, φ(2)= 218.9 (7)° (Cremer & Pople, 1975). The packing is stabilized by weak C—H···π interactions involving the phenyl attached to the nitrogen of the oxazole and the phenyl of the indole fragment (Table 1: *Cg*1is the centroid of the C21—C26 ring and *Cg*2 is the centroid of the C3—C8 ring).

### Experimental

2-Methylene-1,3,3-trimethylindoline (1) is a commercial product. *C*-phenyl, *N*-diphenylnitrone (2) was synthesized according to the literature procedure (Brüning *et al.*, 1973). A solution of (2) (1 g, 6 mmol), (1) (1,12 g, 6 mmol) in ethylacetate (40 ml) was stirred at reflux for 24 h. The solvent was then evaporated under reduced pressure. The residue was crystallized from ethanol, leading to a mixture of diastereiosomers (3) and (3'). They were separated and purified by chromatography on silica gel (eluant: dichloromethane / hexane: 10 / 90). The spirocompounds were finally recrystallized from dichloromethane.

### Refinement

All H atoms were fixed geometrically and treated as riding on their parent atoms with C—H = 0.98 Å (methyl), 0.99 Å (methylene), 1.0 Å (methine) and 0.95 Å (aromatic) with *U*_iso_(H) = 1.2*U*_eq_(aromatic, methylene and methine) or *U*_iso_(H) = 1.5*U*_eq_(methyl).

In the absence of significant anomalous scattering, the absolute structure could not be reliably determined and the Friedel pairs were merged and any references to the Flack parameter were removed.

### Crystal data

**Table:**

C_25_H_26_N_2_O	*F*(000) = 792
*M**_r_* = 370.48	*D*_x_ = 1.225 Mg m^−^^3^
Orthorhombic, *P**n**a*2_1_	Mo *K*α radiation, λ = 0.71073 Å
Hall symbol: P 2c -2n	Cell parameters from 8000 reflections
*a* = 18.0393 (18) Å	θ = 1.7–26.2°
*b* = 8.9854 (7) Å	µ = 0.08 mm^−^^1^
*c* = 12.3947 (9) Å	*T* = 180 K
*V* = 2009.1 (3) Å^3^	Prism, colorless
*Z* = 4	0.48 × 0.36 × 0.28 mm

### Data collection

**Table:**

Stoe IPDS diffractometer	1581 reflections with *I* > 2σ(*I*)
Radiation source: fine-focus sealed tube	*R*_int_ = 0.059
graphite	θ_max_ = 26.0°, θ_min_ = 2.5°
φ scans	*h* = −22→22
19030 measured reflections	*k* = −11→11
2021 independent reflections	*l* = −14→14

### Refinement

**Table:**

Refinement on *F*^2^	Primary atom site location: structure-invariant direct methods
Least-squares matrix: full	Secondary atom site location: difference Fourier map
*R*[*F*^2^ > 2σ(*F*^2^)] = 0.042	Hydrogen site location: inferred from neighbouring sites
*wR*(*F*^2^) = 0.105	H-atom parameters constrained
*S* = 1.15	*w* = 1/[σ^2^(*F*_o_^2^) + (0.061*P*)^2^] where *P* = (*F*_o_^2^ + 2*F*_c_^2^)/3
2021 reflections	(Δ/σ)_max_ = 0.007
256 parameters	Δρ_max_ = 0.26 e Å^−^^3^
1 restraint	Δρ_min_ = −0.15 e Å^−^^3^

### Special details

**Table:**

Experimental. The data were collected on a Stoe Imaging Plate Diffraction System (*IPDS*). The crystal-to-detector distance was 70 mm. 167 frames (4 min per frame) were obtained with 0 < φ < 250.5° and with the crystals rotated through 1.5° in φ. Coverage of the unique set was over 97.4% complete to at least 26.04°. Crystal decay was monitored by measuring 200 reflections per frame.
Geometry. All e.s.d.'s (except the e.s.d. in the dihedral angle between two l.s. planes) are estimated using the full covariance matrix. The cell e.s.d.'s are taken into account individually in the estimation of e.s.d.'s in distances, angles and torsion angles; correlations between e.s.d.'s in cell parameters are only used when they are defined by crystal symmetry. An approximate (isotropic) treatment of cell e.s.d.'s is used for estimating e.s.d.'s involving l.s. planes.
Refinement. Refinement of *F*^2^ against ALL reflections. The weighted *R*-factor *wR* and goodness of fit *S* are based on *F*^2^, conventional *R*-factors *R* are based on *F*, with *F* set to zero for negative *F*^2^. The threshold expression of *F*^2^ > σ(*F*^2^) is used only for calculating *R*-factors(gt) *etc*. and is not relevant to the choice of reflections for refinement. *R*-factors based on *F*^2^ are statistically about twice as large as those based on *F*, and *R*- factors based on ALL data will be even larger.

### Fractional atomic coordinates and isotropic or equivalent isotropic displacement parameters (Å^2^)

**Table:**

	*x*	*y*	*z*	*U*_iso_*/*U*_eq_	
C1	0.43438 (17)	0.9280 (3)	0.0510 (3)	0.0360 (7)	
C2	0.52004 (18)	0.9356 (3)	0.0701 (3)	0.0398 (7)	
C3	0.53941 (16)	1.0776 (3)	0.0091 (3)	0.0346 (7)	
C4	0.60643 (18)	1.1346 (4)	−0.0231 (3)	0.0470 (8)	
H4	0.6508	1.0802	−0.0111	0.056*	
C5	0.6086 (2)	1.2734 (4)	−0.0738 (3)	0.0523 (9)	
H5	0.6547	1.3141	−0.0964	0.063*	
C6	0.5439 (2)	1.3510 (4)	−0.0909 (3)	0.0482 (9)	
H6	0.5460	1.4452	−0.1256	0.058*	
C7	0.47568 (18)	1.2952 (3)	−0.0588 (3)	0.0412 (7)	
H7	0.4312	1.3493	−0.0710	0.049*	
C8	0.47495 (15)	1.1576 (3)	−0.0082 (3)	0.0327 (7)	
C9	0.34450 (16)	0.7260 (3)	0.0744 (3)	0.0363 (7)	
H9	0.2955	0.7656	0.0500	0.044*	
C10	0.38766 (19)	0.8462 (4)	0.1354 (3)	0.0458 (8)	
H10A	0.3531	0.9161	0.1713	0.055*	
H10B	0.4199	0.8006	0.1908	0.055*	
C21	0.36716 (14)	0.6294 (3)	−0.1120 (2)	0.0294 (6)	
C22	0.40480 (18)	0.6491 (3)	−0.2083 (3)	0.0367 (7)	
H22	0.4453	0.7164	−0.2117	0.044*	
C23	0.38362 (18)	0.5708 (3)	−0.2998 (3)	0.0429 (7)	
H23	0.4091	0.5856	−0.3660	0.051*	
C24	0.32514 (17)	0.4710 (4)	−0.2943 (3)	0.0438 (8)	
H24	0.3110	0.4163	−0.3565	0.053*	
C25	0.28787 (18)	0.4513 (4)	−0.1993 (3)	0.0414 (8)	
H25	0.2482	0.3821	−0.1959	0.050*	
C26	0.30746 (15)	0.5317 (3)	−0.1074 (3)	0.0352 (7)	
H26	0.2803	0.5199	−0.0423	0.042*	
C91	0.33383 (16)	0.5862 (3)	0.1405 (3)	0.0359 (7)	
C92	0.39087 (17)	0.4871 (3)	0.1574 (3)	0.0418 (8)	
H92	0.4380	0.5056	0.1259	0.050*	
C93	0.3806 (2)	0.3610 (4)	0.2195 (3)	0.0511 (9)	
H93	0.4205	0.2936	0.2302	0.061*	
C94	0.3131 (2)	0.3330 (4)	0.2658 (3)	0.0537 (9)	
H94	0.3064	0.2460	0.3082	0.064*	
C95	0.2549 (2)	0.4299 (4)	0.2513 (3)	0.0532 (9)	
H95	0.2083	0.4109	0.2841	0.064*	
C96	0.26512 (18)	0.5563 (4)	0.1878 (3)	0.0462 (8)	
H96	0.2249	0.6229	0.1767	0.055*	
C111	0.33893 (17)	1.1202 (4)	0.0002 (4)	0.0526 (9)	
H11A	0.3290	1.2248	0.0175	0.079*	
H11B	0.3032	1.0567	0.0380	0.079*	
H11C	0.3342	1.1051	−0.0778	0.079*	
C211	0.5371 (2)	0.9600 (4)	0.1901 (3)	0.0531 (9)	
H21A	0.5894	0.9871	0.1986	0.080*	
H21B	0.5270	0.8682	0.2301	0.080*	
H21C	0.5058	1.0403	0.2181	0.080*	
C212	0.5604 (2)	0.7992 (4)	0.0285 (4)	0.0576 (11)	
H21D	0.5484	0.7845	−0.0479	0.086*	
H21E	0.5448	0.7117	0.0698	0.086*	
H21F	0.6139	0.8134	0.0366	0.086*	
N1	0.41386 (13)	1.0819 (2)	0.0341 (2)	0.0369 (6)	
N2	0.39329 (13)	0.7023 (2)	−0.0180 (2)	0.0323 (6)	
O1	0.41789 (11)	0.8501 (2)	−0.04964 (17)	0.0370 (5)	

### Atomic displacement parameters (Å^2^)

**Table:**

	*U*^11^	*U*^22^	*U*^33^	*U*^12^	*U*^13^	*U*^23^
C1	0.0486 (17)	0.0268 (14)	0.0327 (18)	−0.0061 (13)	0.0006 (14)	−0.0026 (13)
C2	0.0508 (18)	0.0289 (15)	0.040 (2)	0.0018 (13)	−0.0082 (15)	0.0004 (13)
C3	0.0405 (16)	0.0306 (15)	0.0327 (19)	−0.0027 (12)	0.0001 (13)	0.0003 (12)
C4	0.0412 (17)	0.0488 (19)	0.051 (2)	−0.0023 (14)	0.0057 (15)	−0.0044 (17)
C5	0.061 (2)	0.052 (2)	0.045 (2)	−0.0220 (17)	0.0183 (17)	−0.0051 (16)
C6	0.075 (2)	0.0369 (18)	0.033 (2)	−0.0124 (16)	0.0080 (16)	−0.0010 (14)
C7	0.0545 (18)	0.0294 (16)	0.040 (2)	−0.0016 (13)	−0.0020 (15)	0.0007 (14)
C8	0.0393 (15)	0.0278 (15)	0.0308 (17)	−0.0033 (11)	0.0016 (13)	−0.0016 (12)
C9	0.0392 (16)	0.0353 (15)	0.0344 (19)	−0.0024 (13)	0.0062 (13)	−0.0029 (12)
C10	0.0589 (19)	0.0389 (17)	0.040 (2)	−0.0125 (14)	0.0075 (16)	−0.0062 (15)
C21	0.0326 (14)	0.0234 (13)	0.0322 (18)	0.0021 (11)	−0.0030 (12)	−0.0014 (12)
C22	0.0439 (16)	0.0333 (15)	0.0328 (19)	−0.0024 (12)	0.0017 (13)	0.0000 (13)
C23	0.0537 (18)	0.0432 (17)	0.0318 (18)	0.0047 (14)	0.0002 (15)	−0.0054 (15)
C24	0.0491 (18)	0.0433 (17)	0.039 (2)	0.0008 (13)	−0.0094 (16)	−0.0094 (15)
C25	0.0419 (16)	0.0399 (16)	0.042 (2)	−0.0044 (13)	−0.0055 (15)	−0.0063 (15)
C26	0.0323 (14)	0.0364 (15)	0.0369 (18)	−0.0013 (12)	−0.0045 (13)	0.0005 (14)
C91	0.0445 (16)	0.0323 (15)	0.0307 (17)	−0.0099 (13)	0.0010 (14)	−0.0021 (13)
C92	0.0427 (16)	0.0409 (17)	0.042 (2)	−0.0040 (13)	−0.0009 (14)	−0.0015 (15)
C93	0.061 (2)	0.0437 (19)	0.048 (2)	−0.0014 (15)	−0.0055 (17)	0.0017 (16)
C94	0.066 (2)	0.0429 (19)	0.052 (2)	−0.0193 (17)	−0.0025 (18)	0.0060 (17)
C95	0.054 (2)	0.058 (2)	0.047 (2)	−0.0182 (18)	0.0122 (17)	0.0016 (18)
C96	0.0472 (17)	0.0453 (18)	0.046 (2)	−0.0044 (14)	0.0023 (16)	−0.0026 (16)
C111	0.0396 (17)	0.0475 (18)	0.071 (3)	0.0017 (14)	−0.0049 (18)	−0.0098 (18)
C211	0.066 (2)	0.0489 (19)	0.045 (2)	−0.0085 (16)	−0.0146 (18)	0.0061 (17)
C212	0.055 (2)	0.0375 (18)	0.081 (3)	0.0098 (16)	−0.0048 (19)	−0.0012 (18)
N1	0.0374 (13)	0.0269 (12)	0.0464 (17)	−0.0029 (11)	0.0025 (11)	−0.0012 (12)
N2	0.0410 (13)	0.0248 (12)	0.0311 (15)	−0.0095 (10)	0.0016 (10)	−0.0016 (11)
O1	0.0570 (13)	0.0239 (10)	0.0302 (12)	−0.0113 (8)	0.0029 (10)	−0.0018 (9)

### Geometric parameters (Å, °)

**Table:**

C1—N1	1.446 (4)	C23—C24	1.386 (5)
C1—O1	1.461 (4)	C23—H23	0.9500
C1—C10	1.531 (5)	C24—C25	1.367 (5)
C1—C2	1.565 (4)	C24—H24	0.9500
C2—C212	1.516 (5)	C25—C26	1.395 (4)
C2—C3	1.523 (4)	C25—H25	0.9500
C2—C211	1.534 (5)	C26—H26	0.9500
C3—C4	1.372 (4)	C91—C92	1.377 (4)
C3—C8	1.384 (4)	C91—C96	1.397 (4)
C4—C5	1.397 (5)	C92—C93	1.382 (5)
C4—H4	0.9500	C92—H92	0.9500
C5—C6	1.376 (5)	C93—C94	1.369 (6)
C5—H5	0.9500	C93—H93	0.9500
C6—C7	1.388 (5)	C94—C95	1.375 (6)
C6—H6	0.9500	C94—H94	0.9500
C7—C8	1.387 (4)	C95—C96	1.393 (5)
C7—H7	0.9500	C95—H95	0.9500
C8—N1	1.397 (4)	C96—H96	0.9500
C9—N2	1.460 (4)	C111—N1	1.457 (4)
C9—C91	1.512 (4)	C111—H11A	0.9800
C9—C10	1.531 (4)	C111—H11B	0.9800
C9—H9	1.0000	C111—H11C	0.9800
C10—H10A	0.9900	C211—H21A	0.9800
C10—H10B	0.9900	C211—H21B	0.9800
C21—C22	1.384 (4)	C211—H21C	0.9800
C21—C26	1.390 (4)	C212—H21D	0.9800
C21—N2	1.417 (4)	C212—H21E	0.9800
C22—C23	1.388 (5)	C212—H21F	0.9800
C22—H22	0.9500	N2—O1	1.454 (3)
			
N1—C1—O1	106.4 (2)	C25—C24—H24	120.0
N1—C1—C10	114.6 (3)	C23—C24—H24	120.0
O1—C1—C10	104.0 (2)	C24—C25—C26	120.8 (3)
N1—C1—C2	103.5 (2)	C24—C25—H25	119.6
O1—C1—C2	110.6 (2)	C26—C25—H25	119.6
C10—C1—C2	117.4 (3)	C21—C26—C25	119.3 (3)
C212—C2—C3	113.4 (3)	C21—C26—H26	120.3
C212—C2—C211	110.5 (3)	C25—C26—H26	120.3
C3—C2—C211	108.4 (3)	C92—C91—C96	118.4 (3)
C212—C2—C1	112.8 (3)	C92—C91—C9	121.6 (3)
C3—C2—C1	100.8 (2)	C96—C91—C9	120.0 (3)
C211—C2—C1	110.6 (3)	C91—C92—C93	120.9 (3)
C4—C3—C8	120.1 (3)	C91—C92—H92	119.5
C4—C3—C2	131.2 (3)	C93—C92—H92	119.5
C8—C3—C2	108.6 (3)	C94—C93—C92	120.2 (3)
C3—C4—C5	119.3 (3)	C94—C93—H93	119.9
C3—C4—H4	120.4	C92—C93—H93	119.9
C5—C4—H4	120.4	C93—C94—C95	120.5 (3)
C6—C5—C4	119.9 (3)	C93—C94—H94	119.8
C6—C5—H5	120.1	C95—C94—H94	119.8
C4—C5—H5	120.1	C94—C95—C96	119.3 (3)
C5—C6—C7	121.7 (3)	C94—C95—H95	120.3
C5—C6—H6	119.2	C96—C95—H95	120.3
C7—C6—H6	119.2	C95—C96—C91	120.7 (3)
C8—C7—C6	117.4 (3)	C95—C96—H96	119.7
C8—C7—H7	121.3	C91—C96—H96	119.7
C6—C7—H7	121.3	N1—C111—H11A	109.5
C3—C8—C7	121.7 (3)	N1—C111—H11B	109.5
C3—C8—N1	110.6 (3)	H11A—C111—H11B	109.5
C7—C8—N1	127.7 (3)	N1—C111—H11C	109.5
N2—C9—C91	112.4 (2)	H11A—C111—H11C	109.5
N2—C9—C10	100.6 (2)	H11B—C111—H11C	109.5
C91—C9—C10	112.6 (3)	C2—C211—H21A	109.5
N2—C9—H9	110.3	C2—C211—H21B	109.5
C91—C9—H9	110.3	H21A—C211—H21B	109.5
C10—C9—H9	110.3	C2—C211—H21C	109.5
C1—C10—C9	106.4 (3)	H21A—C211—H21C	109.5
C1—C10—H10A	110.5	H21B—C211—H21C	109.5
C9—C10—H10A	110.5	C2—C212—H21D	109.5
C1—C10—H10B	110.5	C2—C212—H21E	109.5
C9—C10—H10B	110.5	H21D—C212—H21E	109.5
H10A—C10—H10B	108.6	C2—C212—H21F	109.5
C22—C21—C26	119.7 (3)	H21D—C212—H21F	109.5
C22—C21—N2	119.1 (2)	H21E—C212—H21F	109.5
C26—C21—N2	121.0 (3)	C8—N1—C1	108.5 (2)
C21—C22—C23	120.3 (3)	C8—N1—C111	120.6 (3)
C21—C22—H22	119.8	C1—N1—C111	120.4 (2)
C23—C22—H22	119.8	C21—N2—O1	107.6 (2)
C24—C23—C22	119.8 (3)	C21—N2—C9	120.8 (2)
C24—C23—H23	120.1	O1—N2—C9	105.2 (2)
C22—C23—H23	120.1	N2—O1—C1	105.6 (2)
C25—C24—C23	120.0 (3)		

### Hydrogen-bond geometry (Å, °)

**Table:**

*D*—H···*A*	*D*—H	H···*A*	*D*···*A*	*D*—H···*A*
C7—H7···Cg1^i^	0.95	2.89	3.735 (3)	149
C23—H23···Cg2^ii^	0.95	2.95	3.803 (4)	150

Symmetry codes: (i) *x*, *y*+1, *z*; (ii) −*x*+1, −*y*+2, *z*−1/2.
